# Experiences From Developing Software for Large X-Ray Crystallography-Driven Protein-Ligand Studies

**DOI:** 10.3389/fmolb.2022.861491

**Published:** 2022-04-11

**Authors:** Nicholas M. Pearce, Rachael Skyner, Tobias Krojer

**Affiliations:** ^1^ Department of Chemistry and Pharmaceutical Sciences, VU University Amsterdam, Amsterdam, Netherlands; ^2^ OMass Therapeutics, The Oxford Science Park, Oxford, United Kingdom; ^3^ MAX IV Laboratory, Lund University, Lund, Sweden

**Keywords:** macromolecular crystallography, fragment screening, data management, multi-state modelling, data presentation and analysis

## Abstract

The throughput of macromolecular X-ray crystallography experiments has surged over the last decade. This remarkable gain in efficiency has been facilitated by increases in the availability of high-intensity X-ray beams, (ultra)fast detectors and high degrees of automation. These developments have in turn spurred the development of several dedicated centers for crystal-based fragment screening which enable the preparation and collection of hundreds of single-crystal diffraction datasets per day. Crystal structures of target proteins in complex with small-molecule ligands are of immense importance for structure-based drug design (SBDD) and their rapid turnover is a prerequisite for accelerated development cycles. While the experimental part of the process is well defined and has by now been established at several synchrotron sites, it is noticeable that software and algorithmic aspects have received far less attention, as well as the implications of new methodologies on established paradigms for structure determination, analysis, and visualization. We will review three key areas of development of large-scale protein-ligand studies. First, we will look into new software developments for batch data processing, followed by a discussion of the methodological changes in the analysis, modeling, refinement and deposition of structures for SBDD, and the changes in mindset that these new methods require, both on the side of depositors and users of macromolecular models. Finally, we will highlight key new developments for the presentation and analysis of the collections of structures that these experiments produce, and provide an outlook for future developments.

## Introduction

Modern drug development is an intensely multi-disciplinary exercise that relies on expertise ranging from fundamental biophysics to clinical trials (Kiriiri et al., 2020). Especially in the early stages of small molecule development, structural biology has been instrumental in guiding the rational development of numerous novel small molecule drugs (Maveyraud and Mourey, 2020). Structural knowledge of the interaction of a protein with varied small molecule ligands is used to inform the design process of compounds with improved binding affinities (Hughes et al., 2011). Determination of protein-ligand structures is done by X-ray crystallography (Maveyraud and Mourey, 2020), NMR (Nitsche and Otting, 2018), Cryo-EM (Renaud et al., 2018), and even recently with MicroED (Clabbers et al., 2020). However, despite recent breakthroughs in other methodologies (most notably cryoEM), X-ray crystallography remains the workhorse for structure-based drug design (SBDD), at least for the time being.

The prevalence of crystallography in these efforts is a testament to the platform methodologies that have been developed to enable routine crystal structure determination. Over the last decade, macromolecular crystallography has seen a remarkable gain in efficiency and throughput driven by improvements in beam intensity and X-ray detectors, the availability of fast and reliable sample changers, and advances in software for data acquisition and analysis (Owen et al., 2016; Förster and Schulze-Briese, 2019). This has culminated in the development of fully automated beamlines that reduce interaction of the scientist with the diffraction experiment to the barest minimum (Svensson et al., 2019). Moreover, so-called “unattended” data collection in combination with automated data processing (Winter, 2010; Vonrhein et al., 2011; Sparta et al., 2016) and refinement pipelines (Sharff et al., 2011; Wojdyr et al., 2013; Echols et al., 2014; Schiebel et al., 2016) running on high-performance computing systems means that it only takes minutes to get from data collection to high-quality electron density maps. Often overlooked in enabling these achievements is the role and importance of sophisticated and robust systems for managing sample logistics, which are now available at most synchrotron sites (Delagenière et al., 2011). When combined with the technical experimental advances, it is these information management systems which truly enable the establishment of routine high-throughput crystallographic experiments (Zheng et al., 2014).

These advancements have culminated in the establishment of several publicly accessible centers for crystal-based fragment screening (Lima et al., 2020; Cornaciu et al., 2021; Douangamath et al., 2021; Wollenhaupt et al., 2021; Kaminski et al., 2022). The extremes of these setups have now transformed protein crystallography from a structure determination method into another biophysical screening assay technique (Douangamath et al., 2020; Schuller et al., 2021; Günther et al., 2021). These facilities have developed several bespoke software solutions for data capture, processing and deposition, incorporating new software packages for restraints generation for new small molecule compounds, model building and refinement (Sparta et al., 2016; Krojer et al., 2017; Long et al., 2017; Pearce et al., 2017c; Cornaciu et al., 2021; Lima et al., 2021), as well as significant advancements in algorithms for detecting (weakly) binding ligands (Pearce et al., 2017b) and analyzing the output chemical information (Deane et al., 2017). Some of these solutions are still confined to specialized screening setups and are thus only used by a small number of protein crystallographers—some because they are not generic enough and some because they are simply unknown.

Here, we provide an overview of the recent history of software tools for large-scale structure determination and data analysis. Based on experiences from fragment screening, we identify a subset of these approaches which we think deserve closer attention and broader awareness within the structure determination community, and ultimately should be incorporated into the main-stream crystallographic toolbox. Furthermore, we highlight recent developments for analyzing protein-ligand structures that bring together structural biologists and computational chemists.

## Batch Data Processing and Refinement Tools

Currently, the majority of crystallographic structure determination is done through the graphical interfaces of the CCP4 and PHENIX packages (Echols et al., 2012; Potterton et al., 2018). These offer user-friendly interfaces that guide newcomers as well as experienced users through the structure determination process, and both have large (and overlapping) user bases. However, these fundamentally adhere to a “one structure per project” paradigm, where one data set leads to one atomic model which leads to one deposition in the Protein Data Bank (PDB) (Berman et al., 2000). This is suitable for the determination of (a small number of) novel crystal structures, where each chemically-distinct structure becomes its own project, but far less so for the determination of many related-but-distinct protein-ligand complexes, which can now involve the simultaneous determination of hundreds of structures (Schuller et al., 2021). Moreover, the graphical user interfaces have historically lacked adequate meta-data-tracking functionality and provide no direct connections between the various experimental and computational stages of the experiment—neither cloning, expression, purification, crystallization nor data collection. In reality, and in the absence of established, widely-used and integrated solutions that remove the burden of record keeping, many—if not most—practitioners still record and track their experiments in ad-hoc electronic spreadsheets and physical notebooks.

**FIGURE 1:**
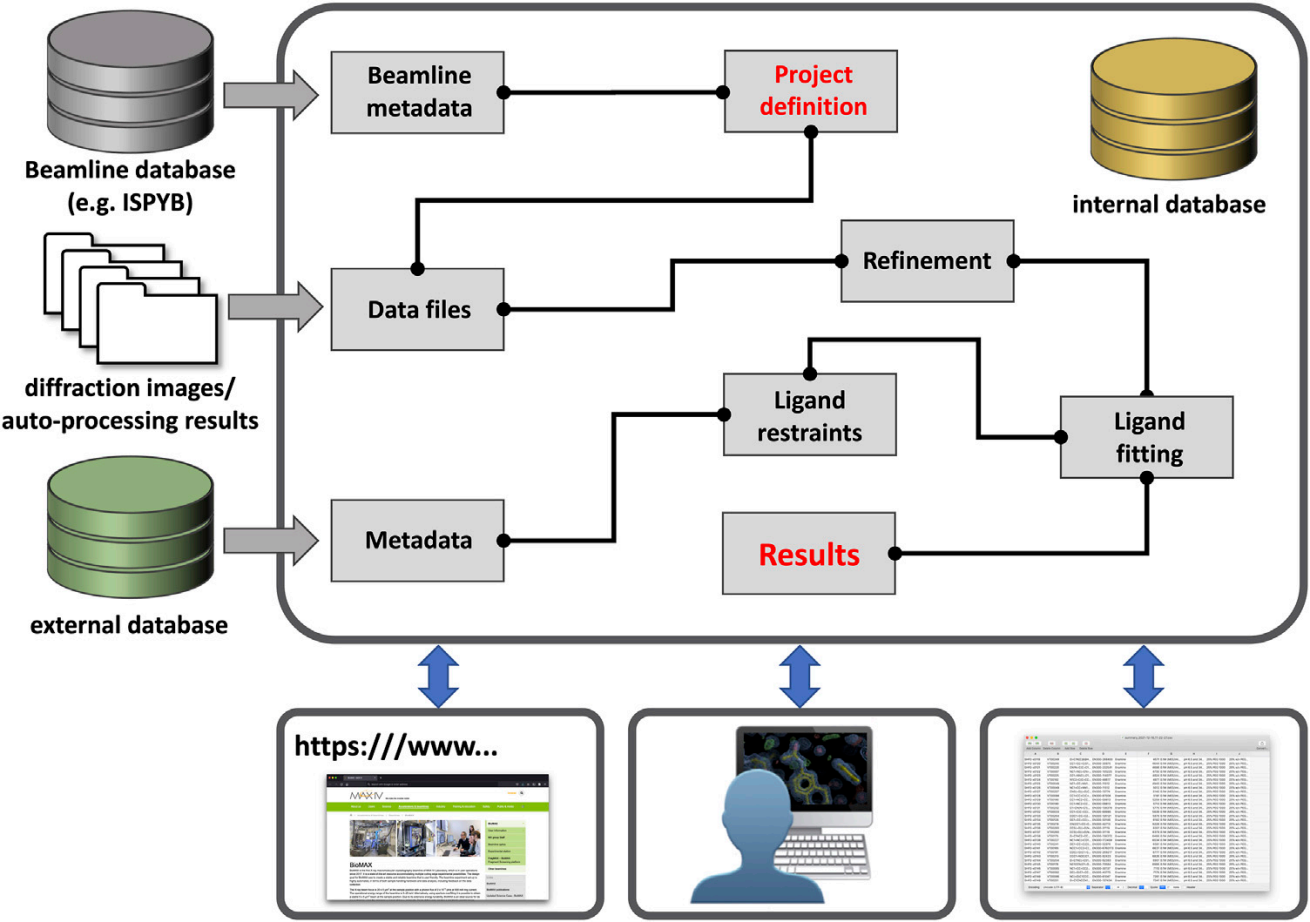
Outline of a generic data processing workbench. Future workbenches will likely be hosted in the cloud [e.g., European Open Science Cloud (EOSC)] and take in various (meta-) data through customizable entry points. Users can then define the sample/data relationship and connect pre-defined tasks as needed for their workflow, such as in KNIME workflows. Workflows could be saved and shared with any other interested party. All results and workflows would be stored in an internal database for each project and local programs or web-services can gain access through a dedicated “Results” node.

The lack of suitable large-scale (publicly-available) processing tools became painfully apparent with the establishment of dedicated synchrotron-based centers for crystallographic fragment screening where hundreds of crystals of the same protein are determined in complex with different compounds (Collins et al., 2017; Lima et al., 2020; Cornaciu et al., 2021; Douangamath et al., 2021; Sharpe and Wojdyla, 2021; Wollenhaupt et al., 2021; Kaminski et al., 2022). As a result, three novel programs—CRIMS, FragMAXapp, and XChemExplorer—became available, which are able to process hundreds of related datasets as part of a single session (Krojer et al., 2017; Cornaciu et al., 2021; Lima et al., 2021; Wollenhaupt et al., 2021). These software differ in design, layout, and scope, but all facilitate batch data processing and record a large set of meta-data in a dedicated database system which facilitates project tracking, thereby enabling posterior analysis as well as simplifying PDB deposition (Figure 1).

XChemExplorer is a standalone program developed at the XChem facility at Diamond Light Source for use with fragment screening experiments (Krojer et al., 2017). It enables the selection of auto-processing results, initial refinement, dataset annotation, interactive model building with Coot (Emsley et al., 2010) and refinement with Refmac (Murshudov et al., 2011) or BUSTER (Bricogne et al., 2017). FragMAXapp was jointly developed between the FragMAX facility at the MAX IV synchrotron and the fragment screening facility at Helmholtz-Zentrum Berlin (HZB) and the BESSY synchrotron (Lima et al., 2021; Wollenhaupt et al., 2021); FragMAXapp offers a web-based platform with similar functionalities to XChemExplorer, but with more customization options for diffraction data processing and initial refinement. Both of these software solutions allow a series of data sets to be consolidated and organized into a single project, and provide launchpads for additional processing with programs such as PanDDA (Pearce et al., 2017b). Finally, CRIMS is a web-based platform developed at the European Synchrotron Radiation Facility (ESRF) and is probably the most comprehensive of the three in terms of meta-data tracking and database integration (Cornaciu et al., 2021); this takes advantage of the extensive database infrastructure at the ESRF High-Throughput Crystallization (HTX) facility and can also directly communicate with the ISPYB laboratory information management system that is widely used at synchrotron beamlines (Delagenière et al., 2011). As a demonstration of the importance of these tools, and their future development, they were instrumental in facilitating the rapid solution and availability of numerous protein-ligand structures of several proteins from Severe Acute Respiratory Syndrome Coronavirus 2 (SARS-CoV-2) in the wake of the coronavirus disease (COVID-19) pandemic (Douangamath et al., 2020; Günther et al., 2021; Newman et al., 2021; Schuller et al., 2021; Kozielski et al., 2022). However, availability of these programs is currently restricted to certain synchrotron sites and they do not allow for customized workflow configuration or provide interfaces for definition of new experiments (Moreno-Chicano et al., 2019; Brändén and Neutze, 2021; Schulz et al., 2022).

## Identification, Modeling and Refinement of Binding Molecules

The second problem that is encountered during crystallographic fragment screening experiments—after the processing of the crystallographic data—is the identification of binding molecules. In a non-focussed fragment screen, only a small percentage of fragments are expected to bind at a particular location on the protein surface, but since fragments may bind at any location on the protein surface, manual inspection of the data quickly becomes infeasible, meaning that automated methods are required (Pearce et al., 2017b).

The most popular crystallographic methods for identifying and validating the presence of bound ligands revolve around internal consistency metrics, i.e., metrics which compare the atomic model and the experimental electron density (Echols et al., 2014; Liebschner et al., 2017). These are used both to identify interesting areas of the electron density map, and to validate the atomic models produced: First, “blobs” are identified by calculating one of a variety of difference maps, and then, constructed ligand models are validated by comparing the model to the experimental electron density. For the purposes of modeling, most methods seek to generate an electron density map that is minimally biased towards the refined atomic model, and which is quantified in terms of a robust signal-to-noise ratio to indicate the importance of an electron density feature. Most approaches therefore utilize different flavors of OMIT maps—in particular OMIT difference maps—which provide a measure of the electron density in a region in terms of the global model error (in units of the rmsd-value of the difference map). This is typically done at the 3-rmsd level (also called the 3-sigma level). Atoms in a selected region must be removed to calculate the difference map, and are either replaced by bulk solvent (Bhat and Cohen, 1984; Terwilliger et al., 2008; Pražnikar et al., 2009) or by vacuum (e.g., polder maps) (Vonrhein and Bricogne, 2005; Liebschner et al., 2017).

It is vitally important, and perhaps underappreciated, to understand exactly what the hereby-identified features in difference maps represent. For the interrogated region, these difference maps measure the density at a particular site relative to either vacuum (polder maps) or bulk solvent (other OMIT maps) in units of the error in your model: significant differences (typically those above 3-rmsd) therefore indicate electron density features that very likely are not experimental noise. However, this indication is different to a measure of signal (such as the presence of an unmodelled bound ligand), since density from any molecule(s), e.g., semi-ordered water molecules, may also produce electron density above this noise level—i.e., it is not only ligand binding that produces electron density in these maps. We should generally expect a map such as a polder map to show a significant amount of density for most sites on the protein surface (except in very poorly refined models or at lower resolution), since even semi-ordered solvent is likely more than three noise units higher than vacuum for high resolution data. Care must therefore be taken when using OMIT methods so as not to overinterpret the maps, whose purpose is only to quantify and show clear unbiased density for a region.

However it is common for the presence of any difference density in an OMIT map to be presented as (incontrovertible) evidence for the presence of a ligand, with even clear cases of mismodeling being difficult to refute (Stanfield et al., 2016a; Stanfield et al., 2016b). The presence of difference density in a particular map—of the appropriate shape and size—may even be misconstrued as evidence for binding by even an experienced user, especially when solving multiple structures with a set of small molecules, since at least one molecule is likely to match a putative blob in a binding site purely by chance, due to the molecules’ small sizes and simple shapes.

**FIGURE 2:**
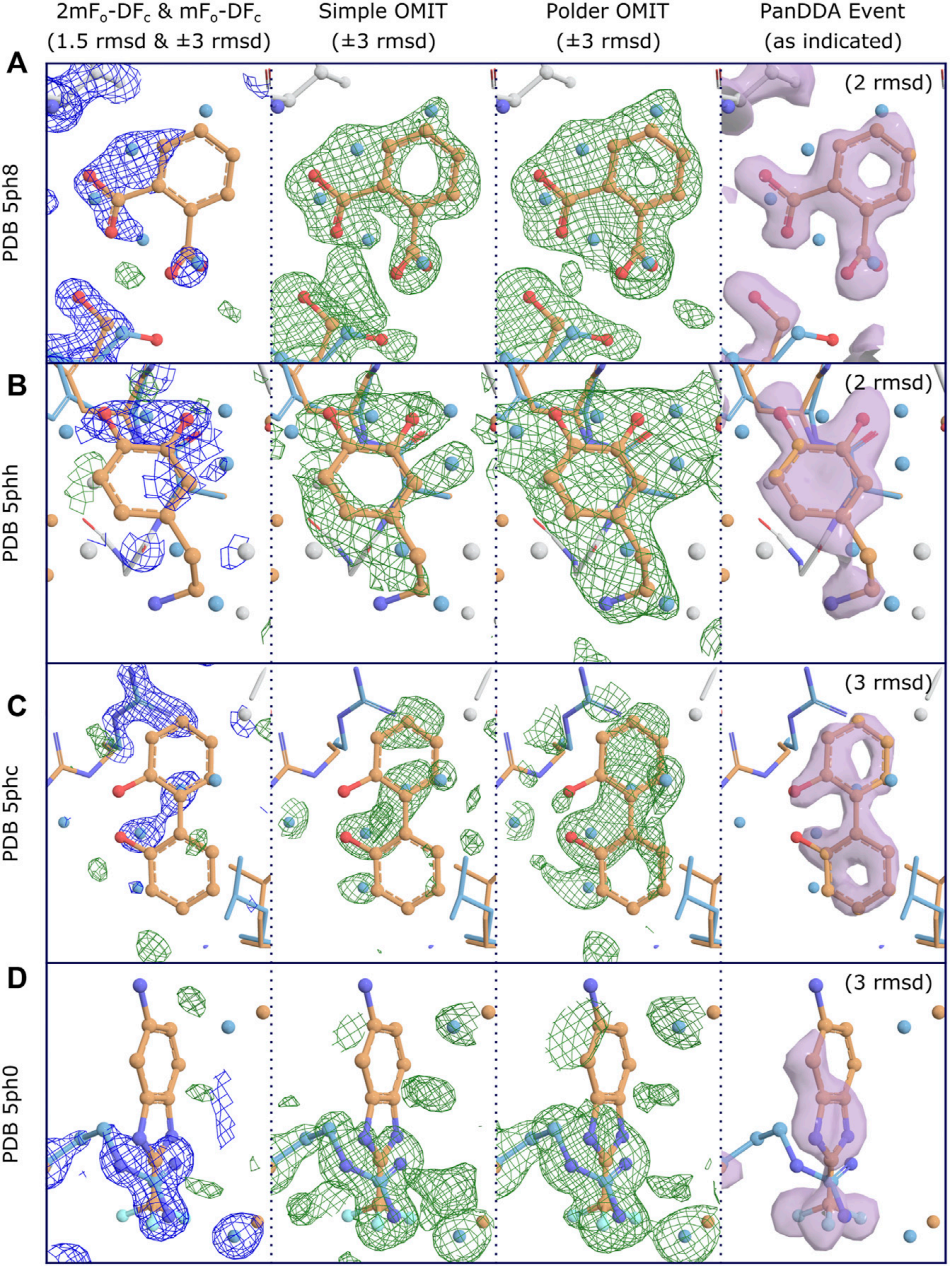
Conventional OMIT maps do not generally produce clear unambiguous evidence of binding for low occupancy ligands, even at high resolution. Examples for four binding fragments showing different levels of support for binding from OMIT maps, where clear evidence of binding is shown by PanDDA event maps. Map coloring: 2mF_o_-DF_c_ maps are shown as blue mesh and all difference maps are shown as green/red mesh; associated PanDDA event maps are shown as purple surfaces. Map type and contour are as indicated. Both types of OMIT maps are produced by phenix.polder. Maps are truncated (carved) at 3Å around relevant residues and ligands for clarity; PanDDA maps are carved at 2Å. Model coloring: To distinguish alternate conformations, carbon atoms for ligand-associated conformations are coloured orange and non-ligand-associated conformations are coloured blue; main-conformation (full-occupancy) atoms are coloured light gray; all other atoms are colored by element except waters, which are coloured as per carbon atoms. Resolutions: **(A)** 1.40Å, **(B)** 1.60Å, **(C)** 1.29Å, **(D)** 1.34Å. Refined ligand occupancies: **(A)** 0.41, **(B)** 0.50, **(C)** 0.38, **(D)** 0.22. PanDDA event map pseudo-occupancies (1-BDC): **(A)** 0.15, **(B)** 0.17, **(C)** 0.10, **(D)** 0.11. **(A)** Binding is not evident in the 2mF_o_-DF_c_ maps at a moderate contour level, but is clearly supported by both types of OMIT map, especially when considered in combination with the extra density from the superposed water molecules from non-ligand-associated conformations, as modeled. It is debatable whether the OMIT maps alone would provide strong enough evidence to support modeling of the ligand, but the single ligand conformation is clearly evidenced in the PanDDA event map, preventing potential misinterpretation of the OMIT map as multiple conformations of the ligand. **(B)** Similar to **(A)**, but with less evidence in the simple OMIT map. The polder OMIT map provides an envelope which fits well with the envelope provided by the ligand and the superposed water molecules, as modeled. It is unlikely either OMIT map would be accepted as evidence of binding, but once more, the ligand conformation is clearly identified in the event map. **(C)** OMIT maps show mostly features which correspond to superposed (not-ligand-associated) waters, and do not present evidence for the bound ligand, unlike the event map. **(D)** Ligand binding coincides with an alternate conformation of an arginine residue, which dominates the refined maps and OMIT maps.

The reason for disagreement about interpretation stems from the nature of OMIT maps. For large strongly-binding ligands, and given medium- to high-resolution data, difference map methods will work well for accurately identifying binding poses, since the correct solution will be well-defined and unambiguous. However, for weakly-binding or smaller compounds, in part due to the reasons described above, these methods are worryingly vulnerable to false positives (incorrectly modeling a ligand which did not bind and is not present in the electron density), as there is no objective approach for determining whether a blob is due to a bound ligand, or due to (semi-) ordered solvent or other molecules in the binding site (Stanfield et al., 2016a; Stanfield et al., 2016b), even when the difference OMIT map shows electron density above a certain threshold. Without robust metrics, what constitutes unambiguity becomes a subjective measure. Opportunities for map misinterpretation are further exacerbated by partial-occupancy ligands, as will be discussed in detail below (Figure 2). Conversely, because the noise level of difference maps is related to the quality of the model, and typical macromolecular atomic models are still generally rather poor (*R*-values of greater than 20% are still the norm), difference maps are not able to identify more weakly binding ligands, since these will fall below the “noise level” of a difference map, leading to false negatives (failing to identify a ligand which did bind).

The large-scale availability of related crystallographic datasets from fragment screening experiments enabled the development of a data-driven multi-dataset ligand identification method: PanDDA (Pearce et al., 2017b). This approach aligns and compares electron density maps from different datasets and identifies local outliers in datasets which deviate from the population of electron density maps; this can be thought of as the multi-dataset generalization of isomorphous-difference (Fo-Fo) maps (Rould and Carter, 2003). For this to work, a number of datasets must not contain binders (i.e., be APO or “ground-state” datasets) against which putative binding datasets can be contrasted. For unfocussed fragment screening experiments, most datasets do not contain a binding fragment, which then constitute APO datasets. Alternately, when performing a fragment screen, a series of true APO datasets can also be collected and used, as is regularly performed at the XChem facility (Douangamath et al., 2021). For datasets with identified outliers, these outliers indicate an “event” at that location in that dataset, which gives strong evidence that a change has occurred in this dataset, and can—in contrast to OMIT maps—be used as a measure of signal. In fragment screening data, events are generally binding ligands, though some random structural changes and processing artifacts can also occur.

An additional insight while developing the PanDDA approach was that the majority of the identified binding events were partial-occupancy features, meaning that the fragment was only bound to a fraction of the protein molecules in the crystal—this is to be routinely expected for fragments, which have low binding affinities. In these cases, which constitute the large majority of identified fragments (Pearce et al., 2017b), the observed electron density is a weighted summation of the electron density for the fragment and also the “apo” or “ground-state” of the crystal, e.g., (dis)ordered water or other solvent molecules. Interpreting the raw experimental electron density map (or any kind of OMIT map) is not feasible in these cases, and indeed should be actively avoided, for fear of misinterpreting the superposed solvent density as part of the ligand density (Figure 2); this could cause mismodelling of the ligand, and would thereby mislead downstream medicinal chemists.

The real power of the PanDDA approach is the overcoming of the partial-occupancy obstacle by estimating the occupancy of the superposed ground state, and subsequently subtracting the appropriate fraction of the ground-state density, which is derived from the analysis of the ground-state (APO) datasets. This subtraction reveals an approximation to the experimental density for the bound state only, i.e., what would be obtained if the ligand was bound at full occupancy. This “event map” can then be used for ligand modeling (Figure 2). Several examples in the original PanDDA manuscript show examples of where interpreting the standard electron density maps lead to misinterpretation, as the standard experimental maps shows diffuse density that could be interpreted as the ligand in multiple conformations; however after application of the PanDDA approach, the ligand is clearly in one conformation, with a superposed solvent state (Pearce et al., 2017a; Pearce et al., 2017b; Pearce et al., 2017c).

In the original PanDDA implementation, no assumptions are made about whether datasets are comparable, and it has since been shown that pre-processing with methods such as cluster4x can dramatically increase the sensitivity of the method even further (Ginn, 2020). Combined, these approaches enable the identification and modeling of very low-affinity compounds which would not have been identified previously, and in doing so have greatly increased the amount of chemical matter available for ligand binding studies. However, the identification of weaker- and weaker-binding ligands have revealed significant weaknesses in currently available approaches for structure determination and usage.

In light of the experiences in fragment screening, it is clear that there is a large and underappreciated potential for misinterpreting electron density when modeling ligands, either because the ligand is not there at all (Stanfield et al., 2016b), or because the ligand binds at subunitary occupancy. Since we cannot know the occupancy of the ligand a priori, we may unwittingly be misinterpreting density for another molecule as density for the ligand, resulting in the wrong pose for (parts of) the ligand (Pearce et al., 2017b). Ligands in the PDB are routinely modeled at full occupancy (Pearce et al., 2017c), showing that partial occupancy is rarely considered. Our experiences with fragment screening have provided numerous cases where the outcome of a PanDDA analysis is very different to what might have been created using traditional approaches (Pearce et al., 2017a; Pearce et al., 2017b; Pearce et al., 2017c), including cases where the ligand was initially perceived to be in multiple conformations, but was in fact in one conformation, and vice versa. The exact prevalence of such mismodelling in the PDB is unknown, and though errors may be minor, inaccurate binding poses of important functional groups could seriously mislead downstream applications such as structure-based drug design.

Since sub-unitary occupancy became an inherent feature of the crystallographic data, this spurred the development of methods for the generation and refinement of multi-state models for superpositions of ligand-bound and ground-state states (Pearce et al., 2017b; Pearce et al., 2017c). Combining the ligand-bound model (derived from the event map) with the ground-state model (derived from the APO data sets) generates a multi-state model for refinement (Pearce et al., 2017c), and combined with appropriate occupancy restraints, this can produce relatively high-quality models for even low-occupancy ligands (Pearce et al., 2017b), although of course these models are lower quality than one would expect from stoichiometric binders. Visual inspection of the refined electron density maps becomes less useful for low-occupancy ligands, since all refined electron density maps will continue to contain multi-state superpositions, and so multiple validation metrics are useful for identifying errant parametric features of the models, such as inappropriate occupancies or B-factors (Pearce et al., 2017b; Pearce et al., 2017c). Iterative modeling using this multi-state approach requires a new mindset for the crystallographer, since it requires the different states of the model to be inspected separately and modeled into different electron density maps (i.e., the ground-state conformation into a ground-state map and the bound-state conformation into an event map), before being recombined for refinement against the original experimental data. This is currently technically difficult, and tools need to be further developed before this can be routinely applied by non-experts. However, routinely ignoring the partial-occupancy nature of ligands in crystallographic models is a significant oversight within the field, the current extent and effect of which is simply unknown.

## Structure Presentation and Analysis

With the influx of large volumes of structural data, such as that generated by synchrotron fragment-screening facilities, application-specific platforms for exploring and exploiting this data become a critical component in the context of fragment-based lead design (FBLD) (Bradley et al., 2015). Popular desktop-based software for the visualizing and analyzing crystal structures (e.g., PyMOL) are only tractable for a handful of structures, and are ineffective in identifying trends in data which could inform further FBLD (Deane et al., 2017). Curating and understanding of FBLD outputs is time-consuming due to the vast number of structures, and often rely heavily on the expertise of the researcher to keep track of interesting and potentially novel features from their analysis (de Souza Neto et al., 2020).

The usage of multi-state models introduces an additional difficulty when using these models in downstream applications. Users of the PDB are mostly not trained crystallographers, and can be confused by the presence of multiple superposed conformations in an atomic model—in the best case scenario, they are simply a nuisance artifact to be removed. Therefore, it is preferable to remove the superposed ground-state conformations from models, and present only the scientifically-interesting bound state. However, the PDB currently has no mechanism for presenting different states of the model, and thus depositors are left with a choice: deposit the full multi-state crystallographic model or deposit only the bound-state model. While the second option—which is the one that has been adopted by the XChem facility—is beneficial to the users of the PDB (who are after all the intended audience), this practice introduces problems for those that wish to reproduce model refinements, since only part of the model has been deposited; this has caused these models to be accurately identified as being poor and unreliable by validation efforts (Wlodawer et al., 2020). Now that hundreds of such models are being deposited in the PDB every year, there is urgently-needed functionality in the PDB for presenting the different components of multi-state models to end users, so that the full crystallographic model can be deposited. The development of routines which further allow the reproduction of PanDDA results are another critical area of required development to ensure that refinements of these models are reproducible and that the models can be validated.

The need for rapid access to 3D structural data necessary for competitive design of lead series in FBLD was brought into sharp focus by the coronavirus pandemic—even once the structures are processed and aligned, annotation and interpretation of this vast amount of information remains a formidable task. A plethora of structural data concerning SARS-CoV-2 and related proteins has been generated, but required a specialized data portal (PDBe-KB Covid-19 portal; https://www.ebi.ac.uk/pdbe/covid-19) (PDBe-KB consortium, 2022) to present data and findings in a coordinated way, including such information as 1) biological function, 2) display of a representative structure, 3) 3D superposed views of the structures and ligands, and 4) lists of relevant publications. Some generic online resources, for example michelaNGLo (Ferla et al., 2020), allow scientists to collaboratively annotate structural data with additional scientific context through an open-source web-based application that allows the creation and sharing of interactive pages containing interactive 3D representations of macromolecular data. Whilst this is extremely powerful, visitors to such pages should be aware that the structural data has often not been officially peer reviewed (though we must also note that peer review of structures in the PDB is not universal). However, since it is well-known that many structures in the PDB are not well-refined or have other serious problems—and that structures are often only made available to reviewers upon request—in both cases it is ultimately still up to individual users to appropriately interpret the data.

**FIGURE 3:**
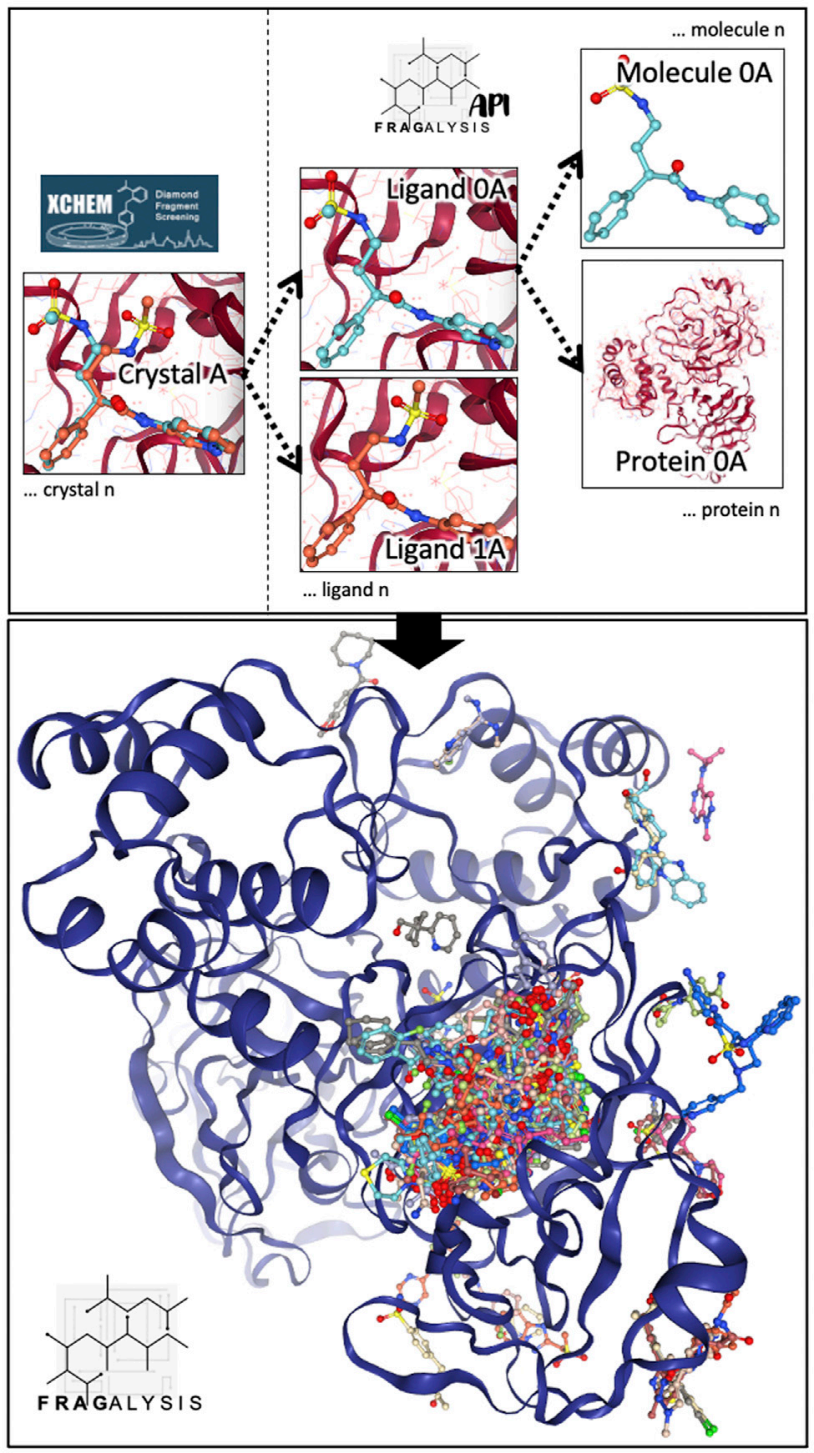
Fragalysis aims to provide immediate access to ligand-protein information without confounding crystallographic artifacts. To achieve this, a given crystal structure (top left—Crystal A) is inspected to find all of the individual ligands. These ligands are then separated into separate bound-state entities (top right—Ligand 0A and Ligand 1A) using the Fragalysis API. Ligands are subsequently separated from their respective protein (top-right Ligand 0A and Protein 0A), and presented in Fragalysis as part of an ensemble of all ligands and proteins in the same reference frame (bottom).

The Fragalysis platform combines the collaborative nature of an online discussion tool with the abilities to interrogate hundreds of crystal structures simultaneously. Fragalysis (https://fragalysis.diamond.ac.uk) is an open-source web-based application which was designed for the dissemination, evaluation and elaboration of fragment screening results from the XChem screening facility (https://www.diamond.ac.uk/Instruments/Mx/Fragment-Screening.html), and is aimed at the non-expert user to facilitate the progression of initial fragment hits from the beamline to more potent protein inhibitors. This is achieved by providing context to crystallographic data: All ligands in each crystal structure are treated as an individual entity, and for each ligand, the protein and ligand can be inspected or analyzed in isolation or together as part of an ensemble (Figure 3). This overlay of structural information provides the user with a starting point to consider how they might apply the existing data to the elaboration and prioritization of new molecules that aim to bind more potently.

In the recent development of Fragalysis, emphasis has been put onto how crystallographers can best communicate the key features and limitations of their crystallographic models to non-expert users, including the presentation of electron density maps—PanDDa event maps, refined 2mF_o_-DF_c_ maps and mF_o_-DF_c_ maps—for newer public datasets (e.g., SARS-CoV-2 Main Protease: https://fragalysis.diamond.ac.uk/viewer/react/preview/target/Mpro), and a new paradigm whereby atoms and bonds in ligands can be highlighlighted and commented upon. This both allows experts to annotate the structures for non-expert users, but also allows users to interrogate the experimental data themselves. These features will be presented fully in future publications. However, it is important to highlight that such developments are imminently necessary more widely in macromolecular crystallography, and structural biology in general. With such a massive amount of data available, and with so many computational methods in FBLD starting from crystallographic models, it is important that the structure-determination community takes responsibility for properly communicating and annotating their data and aiding the wider structural community in how to best interpret and make use of the data beyond crystallographic modeling. Of particular importance is the communication of quality considerations; here, several articles and reviews describe valuable tools for evaluation of raw experimental data and solved macromolecular structures (Kleywegt, 2000; Gore et al., 2012; Adams et al., 2016; Wlodawer, 2017).

Of course, this accumulation and digestion of populations of structures will likely be greatly affected in the near-term by the rapid advances of deep-learning structure-prediction methods such as AlphaFold (Jumper et al., 2021), and RoseTTAFold (Baek et al., 2021). Neither Alphafold nor RoseTTAFold (in their current released versions) incorporate the ability to add native ligands/cofactors, although a cavity which could accommodate one is often observed, nor allow the predicted binding poses of arbitrary ligands, but the development of these functionalities is inevitable and much anticipated.

## Discussion and Conclusion

Despite the gains in efficiency in crystallographic experiments at synchrotrons, driven in part by automated fragment-screening beamlines, there has been no real corresponding increase of X-ray crystal structures in the PDB. The number of yearly released X-ray structures has remained largely the same since 2016 (with a COVID-19-driven exception for 2020). The number of structures that are deposited in the PDB is almost certainly significantly smaller than the real number of datasets collected or structures determined every year, especially since only a small fraction of the vast number of structures determined by pharmaceutical companies ends up in the public domain (Mullard, 2021). Nevertheless, it still takes a remarkably long time to get structures “deposition ready” and then into the PDB. There are two main problems: 1) Structure refinement does not have a clear endpoint and crystallographers often go through many refinement and model rebuilding iterations before they are comfortable depositing their data, and 2) there are hardly any tools available that help with data organization, metadata capture and large-scale PDB deposition. This leads to a situation where crystallographers tend to spend a disproportionate amount of time on finalizing what are usually considered “simple” protein-ligand structures. Consequently, this time is not available for actual structure analysis, which is further exacerbated by a lack of tools for parallel and comparative analysis of related crystal structures, combined with suitable graphical presentation.

Structural biology needs more dedicated and integrated tools for batch data processing, otherwise the gains in experimental efficiency will not result in a corresponding explosion of structural information. CRIMS, FragMAXapp and XChemExplorer begin to tackle these data-organization problems, but they are specialized for the environment where they have been developed, lack flexible workflow configuration, and are restricted to the determination of protein-ligand complexes by X-ray crystallography. While the details still need to be worked out, we would like to outline what such a novel and modular batch-processing workbench could look like. Such a platform should allow flexible and abstract workflow configuration with dedicated APIs for interaction with external databases or processing tools (Figure 1). It should facilitate direct usage of auto-processing results obtained at different synchrotrons and the meta-data stored in databases like ISPYB. This would significantly speed up the process, reduce errors and unburden users from the tedious task of data capture and management of vast amounts of raw diffraction data. Such a tool would not only be useful for single crystal diffraction experiments, but for any other multi-dataset experiment, e.g., serial synchrotron crystallography (SSX) data collections, whether for protein-ligand or time-resolved studies.

Furthermore, there are several paradigmatic lessons for routine macromolecular crystallography to adopt from fragment screening experiments. Our models need to become much more complex than they have been historically, and the databases and visualization methods for presenting these models need to develop quickly to account for this. In the short term, there is still much to be done to convince the community that more complex approaches—i.e., multi-state superpositions—are necessary, but done correctly, more complex structure-determination paradigms should allow for much more robust validation protocols than the current self-consistency metric paradigms. These problems in structure determination and validation are further exacerbated by the sheer number of structures that can be determined, and analytical and visualization methods for identifying trends and common features in these structures are due for an overhaul. Lastly, arguably the greatest responsibility of the macromolecular crystallographic community is to ensure that their data, especially when not proprietary, are released to the public in a timely fashion, and with clear and concise context that make them interpretable to observers and users who may or may not have the necessary expertise to analyze them critically themselves.

These changes will be a great challenge for the developers of databases and software for the deposition, presentation, interpretation and analysis of crystallographic data. These changes should be seen as an opportunity for those developers to redefine how we think about crystallographic data, and to treat this challenge as an exciting new area of scientific research. It is probable that the majority of applications will continue to move towards being web-based, and that more remote computing resources will need to be made available on a global level through academic funding routes. It should also be (made) apparent to funding bodies that disciplines like crystallography don’t function without robust, scalable, and sustainable software behind the scenes, and that more needs to be done to ensure secure medium- and long-term funding for software so that it can be developed and maintained appropriately. This must include funding streams to support individual research software development.

Conversely, it is the responsibility of computational scientists who develop new methods and algorithms to properly explain the relevant applications of their methods, and to ensure that those methods are made available where they are not proprietary. This includes publishing them in a version-controlled environment such as GitHub, and publishing links to these repositories within their manuscripts. It is our opinion that making data and algorithms ‘available upon request’ is no longer good enough, given the availability of a number of easy-to-use public repositories such as Zenodo (European Organization For Nuclear Research and OpenAIRE, 2013), where arbitrary files can be uploaded; to this point, the data and scripts for reproducing the OMIT maps for Figure 2 have been uploaded to Zenodo (https://doi.org/10.5281/zenodo.6334726).

Though the experiences and methods highlighted in this manuscript primarily arose from highly specialized experiments in fragment-based discovery, we believe many of the approaches should inspire developments in other areas of macromolecular crystallography. For instance, the multi-dataset-management approach to experimental crystallography is one that might greatly smooth the process of determining a (set of) typical crystallographic structure(s), and remove much of the tedious meta-data tracking, whilst more technical model-building aspects also have clear applications in more niche crystallographic experiments such as time-resolved crystallography, Laue crystallography, and X-ray Free Electron Laser (XFEL) experiments.

## Data Availability

OMIT map figure data has been uploaded to Zenodo (https://doi.org/10.5281/zenodo.6334726). Further inquiries can be directed to the corresponding author.
